# 

**Published:** 1876-09

**Authors:** 


### TABLE OF CONTENTS.

ORIGINAL COMMUNICATIONS—

Medical Observations During a Tour Through Europe in 1875–'76. By Swan M. Burnett .... 513

SELECTIONS—

Diptheria Successfully Treated. By E. Chenery, M. D., Boston.... 521
Rigidity of the Os Uteri Overcome in Obstetric Practice.... 526
Therapeutic Value of Certain Remedies. By H. E. Woodbury, M. D., Washington, D. C.... 529
Treatment of Purpura.... 532

EXTRACTS AND GLEANINGS—

Cicuta in Scarlet Fever—535. The Electrolytic Treatment of Tumors—536. Electricity, Its Therapeutic Applications—538. Odorless Iodoform—539. Diptheria Treatment—540. Spider Web or Tela Araneæ in Chronic Intermittents—541. The Symptoms and Prognosis of Cerebral Anæmia—542. Gastric Vertigo—543. Amyl Nitrite in Nervous and Mental Diseases—545. Diagnosis of Fissures of the Anus in Children at the Breast—545. Specific against Hydrophobia—546. Treatment of Placenta Prævia—547. New Treatment for Tricophyton—548. The Indications for Quinine in Surgical Practice—548. For Soap Liniment—549. The Plethysmograph—549. Topical Treatment of Chronic Dysentery—550. Intestinal Atony—550. Acute Bright's Disease—Sialagogue Effect of Jaborandi—551. Sulphurous Acid in Enteric Fever—551. Convulsions Arrested by the Sinistro-Lateral Posture—552. Hyposulphite of Soda in Diphtheria—552. Gelseminum—552. Acute Articular Rheumatism—Ice Treatment—553. Liquor Patassæ in Diphtheria—554. Chronic Ulcers of Leg—555. Prophylactic Treatment of Puerpural Fever—556. The Treatment of Pruritus Hiemalis—557. Treatment of Pruritus Vulvæ—558. Atropia as an Antidote to Prussic Acid—558. Therapeutic Value of Picrotoxine—559. Syphilitic Headache and Neuralgia Cured by Small and Repeated Doses of Calomel—560. Action of Jarborandin or Chlorydrate of Pilocarpine—560. Treatment of Epistaxis by the Internal Administration of Ergot—561. Esmarch's Bandage for Chronic Ulcers—561. External Treatment of Urticaria—562. Homicidal Handwriting—562. Pomegranate Bark for Tape-worm—563. Preparations of Iodoform—564. Recipe for Curing a Taste for Liquors—554. Sages Catarrh Remedy—565. Two New Sources of Lead Poisoning—565. Treatment of Diphtheria by Clysters—565. Cholcate of Soda as a Preventive of Gall Stones—566. Bronchocele—Treatment by Injections of Alcohol—566. Photography as a Means of Diagnosis—566. Sulphide of Calcium in Diabetes—566. Powder to Prevent Pitting After Variola—567. Cyanides in Acute Articular Rheumatism Treated with Salicylic Acid—567. Sulphurous Acid as a Dressing for Recent Wounds—568. Incontinence of Urine—568. Meniere's Disease Treated by Quinine—568. Chloroform in Hydrocele—568. Poulticing in Hæmoptyses—568. Coto Bark in Diarrhœa and Rheumatism—569. Treatment of Epistaxis by the Internal Administration of Ergot—569. Cold in the Head—569. Animal Vaccination—569. Epistaxis—569. Cod-Liver Oil Injections—569. The Sialagogue Effect of Jarborandi Prevented by Hypodermic Injection of Morphia and Atropia—570. Toothache—570. Painful Menstruation—570. Chloral in Ozæna—570. Chloral as an Antiseptic—570. Rancid Oil of Maize in Skin Diseases—570. Powder for Producing Ozone—571. Cold Water Bath in Summer Complaint of Children—571. Castor Oil—571. Daturia as a Substitute for Atropia—571. The Action of Coca—572. A Nut for Contagionists—572. Salicylic Mixture in Diphtheria—572. Hæmoptysis Treated by Ergot—572.

EDITORIAL AND MISCELLANEOUS—

En Ethical Puzzle—Two in One.... 573
Yellow Fever at Savannah.... 576
Literary Notices.... 576

